# Implementation of a telemedicine, stroke evaluation service; a qualitative study

**DOI:** 10.1186/s12913-022-08428-x

**Published:** 2022-08-13

**Authors:** Elin Kjelle, Aud Mette Myklebust

**Affiliations:** Department of Optometry, Radiography and Lighting Design, Faculty of Health and Social Sciences, University of South-Eastern Norway, Post office box 235, 3603 Kongsberg, Norway

**Keywords:** Computed tomography, Stroke evaluation, Telemedicine, Organization of health services, Implementation

## Abstract

**Background:**

Acute ischemic stroke requires early medical imaging with a computed tomography (CT) scan and immediate thrombolysis treatment. In rural areas, the long distance to the nearest hospital reduce the patients’ probability of receiving medical assistance within the 4.5-h period. The aim of this study was to assess how the service was set-up, and how managers and personnel experience the organisation and value of a rural telemedicine, remote controlled CT stroke service.

**Methods:**

Ten semi-structured individual interviews and one semi-structured focus group interview were conducted. The sample included 15 participants involved in the telemedicine service in Hallingdal, Norway. The interview guide consisted of questions on the service, experience of working with the service, value and quality, management, and challenges. Interviews were recorded and transcribed verbatim. Thematic content analysis was used to develop a narrative of the findings.

**Results:**

Findings were categorised into three main categories; value of the service, organisation of the project, and from project to permanent service. Participants perceived the service to be valuable for patients and the local community. The service included task shifting from radiographers and junior doctors to the local paramedics. To enable long- term operation of the service the participants suggested management, coordination, and continuous training as important factors.

**Conclusions:**

The service was perceived as valuable to the local community, providing a sense of healthcare security and equitability. Management’s involvement, flexibility, and coordination appears to be a key factor for successful implementation and long-term sustainability of the service.

## Background

Acute stroke is a common condition in high-income countries. Worldwide one in four people over the age of 25 will be affected by a stroke during their lifetime and just in 2016 more than 9.5 million cases of ischemic stroke were reported [1]. Management of acute ischemic stroke has changed during the last years after demonstration of the efficacy of thrombolysis treatment [2]. This therapy has improved the outcome for patients with vessel occlusion; however, treatment must be given within 4.5 h of onset of symptoms [3]. In remote and rural areas, it can be challenging to reach the nearest hospital and start the treatment on time. Telemedicine can assist stroke assessment in prehospital service, however suitable technologies are still developing [4]. In addition, telemedicine can be used to guide on-site personnel with medical imaging and treatment to save patients’ lives or to limit the damage caused by a stroke [5].

In the rural area of Hallingdal in Norway, patients need to travel up to 3 h to reach the nearest local hospital where a stroke healthcare team is available on site. To provide an immediate alternative service for these patients at a nearby medical centre where a Computed tomography (CT) -scanner is available, an ongoing telemedicine implementation project was initiated. The service requires that nights and weekends the on call paramedics perform the CT examination guided by a radiographer at the local hospital, and a junior doctor guides the paramedic to perform a neurological examination (National Institute of Health Stroke Scale (NIHSS)) and start the thrombolysis treatment if appropriate [6]. Kjelle & Myklebust [5] describes the specific procedure for this service in more detail. This type of telemedicine service initiated by Ibsen and Hall [6] is the first implemented in Norway and to the authors’ knowledge novel internationally as well.

Implementing a telemedicine service requires suitable equipment, high bandwidth, and fast routing [7]. In addition, planning, flexibility, and dedicated mangers, coordinators, and personnel are key to its success [8–11]. The Hallingdal stroke evaluation project also required task shifting and changing of routines in several departments and across health care organisations. Staff taking on new or expanded roles formerly performed by other professionals require further training and education [8, 9, 12]. Organisational and system context, as well as the culture in the specific organisation is thus essential for implementing an effective task shifting health service [10, 11, 13]. In this study’s context, the driver for task shifting was a pragmatic response to meet the need for stoke evaluation by use of CT diagnostics, neurologic examination, and thrombolytic treatment.

As part of a larger project assessing the telemedical, stroke evaluation team, the aim of this second study was to assess how the service was set-up, and how managers and personnel experience the organisation and value of a rural telemedicine, remote controlled CT stroke service.

## Methods

### The study context

The Norwegian healthcare system is mainly a public system based on general taxation [14]. Local health services are managed at municipal levels while specialised healthcare, including imaging services, is largely provided by hospital trusts managed on a regional level [14]. The Co-ordination Reform, implemented in 2012 aimed to improve the quality of the health services in a sustainable manner, and improve the proximity of services to patients in rural and remote areas [15]. Accordingly, local medical centres (community hospitals) organised under the hospital trust were established to provide health services for the local population and tourists as a combination of primary and specialised care [16, 17]. In some of these local medical centres, as in Hallingdal, there is an X-ray machine and a CT scanner [17]. Hallingdal is an area of 5.832km^2^ with 20.532 inhabitants, 3.5 people per km^2^ [18]. The area is popular for tourists, and in 2018 there were 3.6 million tourist visits reported [19]. The Hallingdal local medical centre offers health services to both inhabitants and visiting tourists [6]. The travel distance in good driving conditions to a hospital is estimated between 1–3 h depending on location [6]. The location of the local medical centre reduces travel times from 2.5 h to 40 min by ambulance for the most remote areas [6].

### Participants

Ten semi-structured individual interviews and one semi-structured focus group interview were conducted in this qualitative interpretative descriptive approach to assess the participants’ experience of a telemedicine service. The individual interviews included radiographers, paramedics, physicians, hospital managers, and project managers. The focus group included managers from the prehospital services, local health centre, and the municipality. The participants were all experienced in their field with more than 5 years of clinical practice or management experience. In total seven male and eight female participants were interviewed. Details on profession and role in the service are indicated in Table 1.

**Table 1:** An overview of the participants’ profession and role in the service divided by individual and focus group interviews

	Individual interview	Focus group
Paramedic	2 service performers	1 manager
Radiographer	2 service performers	1 service performer
Junior doctor	2 service performers	
Hospital manager	1 physician	
Local medical centre		1 administrative manager
Project manager	2 physicians, 1 radiographer	
Municipal manager		1 administrative, 1 chief physician

The participants in this study were recruited from the local medical centre, municipality, hospital, and pre-hospital service using volunteer sampling [20]. An invitation was sent to the hospital trust and local municipalities asking for candidates in accordance with the inclusion criteria. A list of possible participants was provided and EK and AMM invited candidates to participate via e-mail providing an information letter and consent form. All participants volunteering returned the consent form before an interview was set up. The inclusion criteria were managers, radiographers, paramedics, or physicians who managed or had been involved in performing the remote CT stroke service.

### Data collection

The semi-structured approach was chosen to allow relevant topics to be explored openly, and at the same time ensuring that the same topics were discussed with all participants [21]. An interview guide with open-ended questions was developed. Core issues derived by experience included; competence, training, communication, cooperation and the quality of service. The interview guide is available in Table 2. The individual interviews were conducted in June 2020 and October 2020 online, due to the Covid-19 pandemic, using the Microsoft Skype for Business (2016) software. The focus group interview was conducted face to face at the local medical centre in June 2020. A second focus group with hospital managers was planned but due to the number of recruited participants, it had to be substituted for individual interviews. A Zoom H1 Handy Recorder recorded the dialogue of the interviews. The authors, both female, experienced radiographers working at a university jointly interviewed the participants. Both authors introduced and explained the project to the participants before the interview. The authors were not involved in any procedures of the investigated project. The individual interviews lasted on average 36 min (22–47 min). The focus group interview lasted 52 min. In order to reach consensus [21] at the end of each interview the main interviewer summarised the participant’s statements of the main subjects. The participant could then comment on the summary, clarify misunderstandings, or add details if needed.

**Table 2:** The questions in the interview guides used for this study

Questions for performers	Questions for managers/focus group
What is your experience in working with remote controlled CT stroke evaluation?	What is the value of the remote-controlled CT stroke evaluation in this area?
Why do you think this service is important for the Hallingdal area?	How was the service managed during the project?
How would you rate the quality of this service and how is it functioning?	How does this service affect the quality of the health services in this area?
What challenges are there in the delivery of this service?	Are there any challenges?
	What facilitates continuing this service when the project ends?

### Analysis

Inductive thematic content analysis was used to analyse the data, a well-established approach in thematic analysis of semi-structured interviews [21]. Data from individual and focus group interviews were analysed together. The analysis process consisted of six steps based on the descriptions of content analysis of Graneheim and Lundmann [22] presented in Table 3.

**Table 3:** Description of the steps of thematic content analysis used in this study

Step		Action
1.Transcription	Listening through the recorded interviews and verbatim transcribing the dialogue	EK and AMM transcribed half of the interviews each
2.Familiarization	Reading through all the transcripts to get an overview	Both authors read all the transcripts
3.Coding	Assigning codes to sections of the text inductively to create meaning units, and sorting data by codes	Both authors coded all the transcripts and met to discuss the codes used, EK sorted and colour coded meaning units
4.Condensation	Condensation of meaning units into shorter statements, still using the participants words and phrasing	All meaning units were condensed by EK
5.Categorization	Sorting meaning units into groups that are exhaustive and mutually exclusive. Names of categories are assigned through the content of the meaning units	Condensed meaning units were sorted into subcategories and categories by EK these were reviewed by AMM
6.Themes	Assigning categories into overarching themes	Through discussion both authors together sorted categories into themes

To illustrate how steps 3 to 5 are used on the interview data, an example is given in Table 4.

**Table 4:** An example of how a coded meaning unit is related to categories

Code	Meaning unit	Condensed meaning unit	Subcategory	Category
Want the service to continue	They get a much better service. To save transfer time because the scanner is there. To stop this service now would be indefensible, no matter who pushes the button. (Project manager)	Need to continue the service to give patients the best possible care	Continuing the service	From project to permanent service

### Ethics

The Norwegian Centre for Research Data approved the processing and storage of personal information in this study (Ref. 358,427).

## Results

**Fig. 1:**
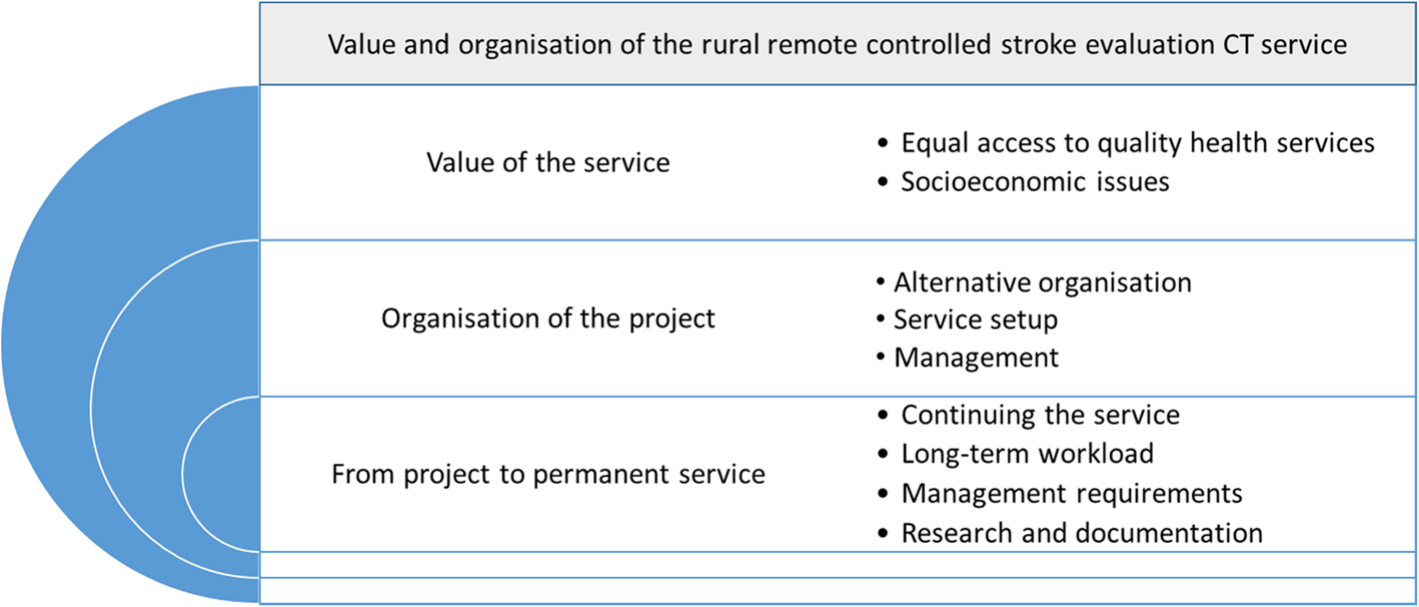
Overview of theme (grey box), main categories, and sub-categories (bullet points)

Based on the participants experience the analysis revealed topics related to value of the service, resources used, the responsibilities in the project, and the progression from project to establishing a permanent service in Hallingdal. Value and organisation of the remote-controlled stroke evaluation CT service was identified as the main theme. Findings were organised into three main categories with two to three sub-categories each presented in Fig. 1. The main categories were “Value of the service”, “Organisation of the project”, and “From project to permanent service”. Each category will be described in detail in separate sections and exemplified using quotes.

### Value of the service

#### Equal access to quality health services

Even though there were no reports or data published from the project all participants experienced having a CT scanner in the area as improving health care quality. They perceived the service as safe and that the inhabitants felt safer knowing that medical assistance was within proximity in the event that themselves or a loved one should suffer a stroke. In addition, the participants experienced that this service was a way to make the health service in rural areas comparable with services provided in urban areas. Provision of timely and equitable healthcare services was viewed as right and just. One municipality manager said: “*This service is very important to our citizens, and the patients are satisfied*.”

Living in rural areas has the disadvantage of longer travel time for patients to reach a hospital to receive high quality care in case of acute stroke. All the participants identified this as the most important problem the service could overcome and thus prevent patients from developing severe complications. In addition, the participants expressed the importance of the patients being able to stay close to family and friends. The travel time does not only affect physical but also psychological burden on patients and their families. For instance, in cases where patients experience a large brain haemorrhage and accompanying complications, the participants explained the benefits of the patients receiving palliative care closer to home and loved ones instead of spending their last hours in an ambulance or at a hospital far away. One project manager said. “*In some patients the bleed is so big and the patient more than 90 years old, then the patient can stay at the local medical centre with his or her family and receive good palliative care rather than spending their last hours in an ambulance*.”

#### Socioeconomic issues

In addition to the value for the individual patient, the participants expressed that the service would have a socioeconomic value. Using the already available personnel on call at both the local medical centre and hospital was considered essential to keep costs to a minimum. The investment in equipment was weighted as a smaller cost compared to keeping extra personnel on call 24 h a day, 7 days a week. In addition, quickly starting thrombolysis reduces complications for the patients, which the participants suggested benefits society as the costs of rehabilitation and hospitalisation are in the long term reduced. One project manager said: “*The CT and the rebuild was 650,000 €, if we save one person from living in a wheelchair for the rest of their life, society have earned back that investment*.”

### Organisation of the project

The project started when a CT scanner was installed in the local medical centre. Initially it was operational during the daytime only as this was when a radiographer was present. However, as the respondents all agreed, stroke can occur at any time of day, and there was need to find a way to keep the CT operational for stroke evaluation during the night and weekend as well. The project wanted to follow the same procedures as performed in hospital, and at daytime in the medical centre. The procedure being that patients with suspected stroke without a visible bleed on the unenhanced CT undergo a head CT angiography (CTA) to assess if there is a thrombus and its location, as this assist to determine the appropriate treatment.

#### Alternative organisation

There were insufficient funds to employ more radiographers in the local medical centre; therefore, a system that uses personnel who are already on call was needed. At the Hallingdal emergency room, a doctor is present 24-h a day. However, training the doctors to conduct the CT-scan and thrombolysis treatment was perceived as complicated. Most of these doctors are foreign, substitute doctors, and the continuity of the training would be hard to achieve. Near the local medical centre there is an air medical service station with doctors on call. However, they cover a large area, and would often be away on assignments. The management decided to train the paramedics on the local base. One project manager said: “*We understood that we needed to use personnel on call, and that was the paramedics.*”

#### Service set-up

In the beginning, the project was planned without directly involving the radiology department. The project initially considered the paramedics capable of operating the CT-scan and the junior doctors with support from a consultant physician to interpret the CT images. Thus, there was limited inclusion of the radiologists in the project. One project manager said. “*We make the diagnostic decision without them [Radiologists]; we informed them about the project, but no specific cooperation*”

However, the radiographers were sceptical regarding paramedics operating the CT-scan independently. The radiation protection regulation states that radiographers must be involved in CT scanning, and thus radiographers needed to be involved in the project. Through the remote-control system, this became possible without employing more radiographers locally. However, not involving the radiographers from the beginning did raise scepticism and doubts as to whether the project managers understood what knowledge was required to operate a CT-scan and the risks to the patient. One project manager said: “*They started the project without us [radiology department]; it became an unnecessary though start*.”

Another challenge was that the radiographers could not perform the contrast tracking needed for a head CTA via the remote-control system, as the system creates a delay. Paramedics preparing and administering contrast media was also observed as a factor complicating and adding time to the procedure with limited gain to patients’ outcome. Thus, the procedure was changed to start thrombolysis first to open the blood supply to the brain cells and if needed, perform the CTA in the local or tertiary hospital later, depending on the patient’s condition. One radiographer said: “*It is not possible to remote control the enhanced scan, as the remote control creates delay. Then the automatic tracker does not function properly*.”

**Fig. 2:**
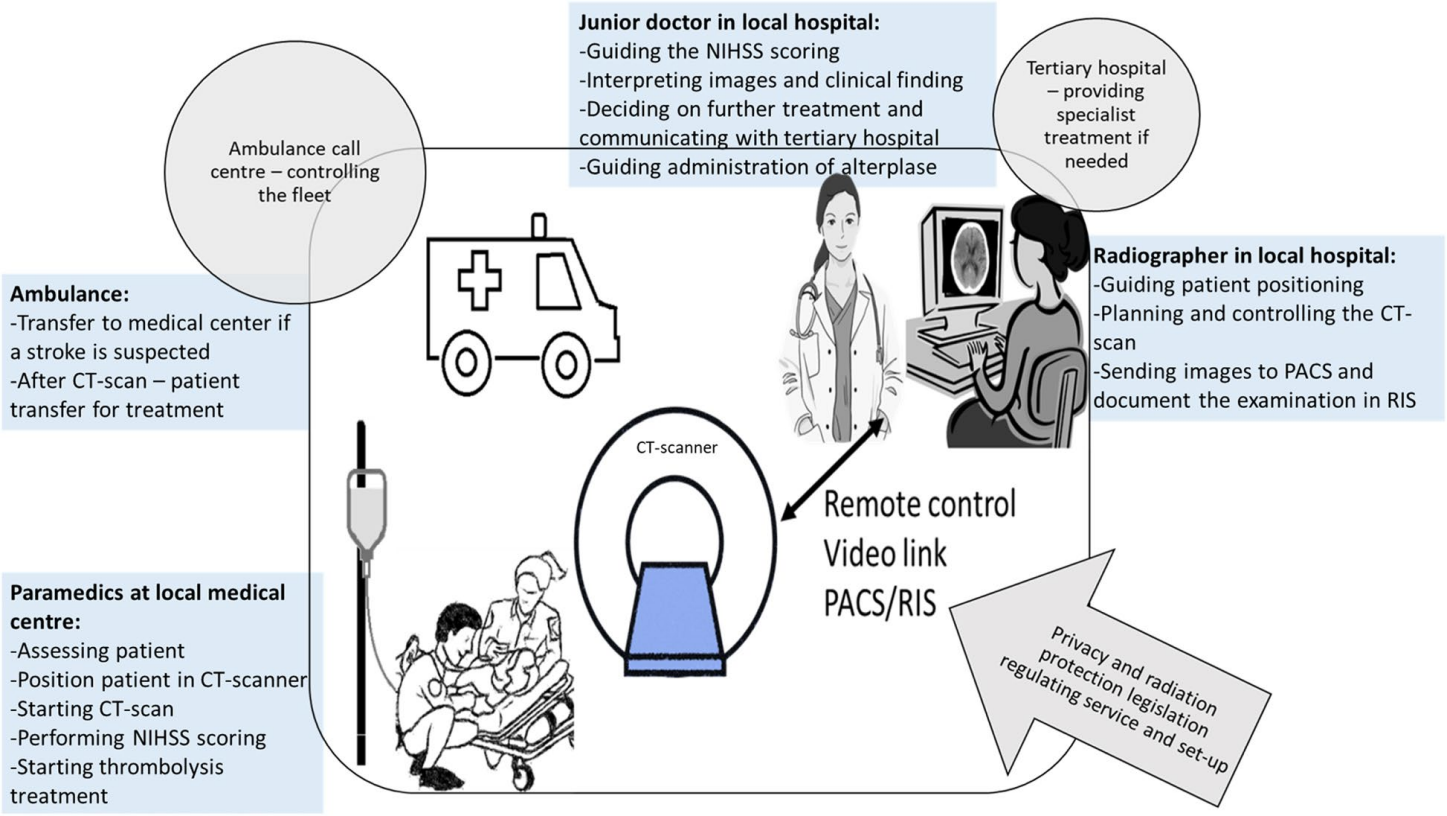
An illustration of the tasks, organisation and equipment needed in the telemedicine remote-controlled CT stroke service in Hallingdal local medical centre. Blue boxes describe tasks, grey circles represent third parties involved in the project, grey arrow illustrates regulation affecting the organisation of the service, and black arrow represent the technology used in the telemedicine service. (NIHSS—National Institute of Health Stroke Scale, PACS—Picture archiving and communication system, RIS – Radiology information system)

After discussions and planning, the service was organised as illustrated in Fig. 2. The CT at the local medical centre was set up for remote control from the local hospital where a radiographer and junior doctor is on call 24-h a day. A video link system in the scanner room is used for the junior doctor to observe the patient and communicate with the paramedics and for the radiographer to observe and guide the positioning of the patient in the scanner. The local medical centre uses the same radiology information system (RIS) and picture archiving and communication system (PACS) as the local hospital, thus the images are stored and viewed in the hospitals PACS.

The paramedics were trained to position the patient in the CT scanner, and start the scan guided by a radiographer. Further, they were trained to do a NIHSS examination and starting thrombolysis supervised by a junior doctor. The ambulance call centre controlling the fleet of ambulances in the area developed special procedures for stroke patients in the Hallingdal area. Securing paramedics included in the project to be present in the area as much as possible. The junior doctor at the hospital communicates with the tertiary hospital in cases where patients are candidates for endovascular thrombectomy. Moreover, the tertiary hospital changes their procedures to accept patient transfer without a head CTA performed.

The service set-up required different governmental approvals. To allow paramedics to CT-scan, the project needed a dispensation from the Norwegian radiation and nuclear safety authority. To obtain approval the paramedics needed education and training in radiation protection. The project radiographer and the radiation protection officer at the local hospital provided education and training. One project manager said: “*We are working under dispensation from the Norwegian radiation and nuclear safety authority.”*

In addition, opening for remote control of the CT scanner rendered the system vulnerable to a privacy or security breach. A risk evaluation had to be conducted to get a privacy approval through the IT-department. This was experienced as a bureaucratic and time-consuming process for the project, and the risk was considered low. One project manager said: “*We needed a risk evaluation and privacy approval for running the remote-control system. That took 6 months; it was a long process for a project with such low risk*.”

#### Management

Close management and evaluation were experienced as important in the project. This project has one main project manager with a supervisor who is also involved in service planning, set-up, and research. In addition, there is a project radiographer in a 20% position in charge of the organisation and training of radiographers and issues related to the CT-scan and image quality. The main project manager is well known among the employees and trusted by all involved personnel and cooperating managers in other departments. This manager is responsible for the education and training of the paramedics and junior doctors and for setting up weekly training sessions and evaluation patient cases. The project manager also takes responsibility for patient safety during the project. One project manager said: “*Yes, I am responsible for the training, and if something goes wrong it is my responsibility*.”

### From project to permanent service

#### Continuing the service

As all the participants experienced this service to be of high value to the patients and the community, there was mutual agreement that the service was necessary and should continue after completion of the project. The managers from the local medical centre and municipalities considered this service vital. Radiographers and junior doctors however expressed concern that the low number of patients per year would be seen as a reason for the hospital to shut down the service when the project ended. One radiographer said: “*As I see the project now, I think it should continue, however it might be the number of patients that decides if that is possible*.”

#### Management requirements

The project managers expressed a need for the ordinary management at the local hospital to be included in the service if it should continue after the project period. From their experience, there is a need for management involvement in both the medical and radiological departments to secure continuous training for paramedics, radiographers, and junior doctors. Moreover, for including new employees as they join the departments. In addition, there is a need for evaluation and follow-up of patient cases and the service organisation and functioning. One project manager said: “*Management needs to decide this; however we need a doctor from the stroke unit to be in charge and organise the training. Now we have a radiographer 20% dedicated to this project and that is essential. We cannot continue the service without a radiography manager dedicated to this service*.”

#### Long-term workload

Involving the on call radiographer led to the radiographers experiencing an increased workload. Before the project started, the radiology department had one radiographer on call in the evenings, nights, and weekends. The radiographers considered dealing with a remote-controlled service in addition to the usual on call work indefensible. Thus, management increased the number of radiographers to two radiographers in the evenings. The radiographers however still express working alone on a night call as a risk. One radiographer said: “*The workload on the radiographers increased, management increased the number of radiographers to two in the evenings; however, at night we are alone. We are working to change this, so that we would always be two radiographers on-call*.”

#### Research and documentation

The service is currently part of a research project assessing patient outcome and cost-effectiveness. That research was regarded by the participants as essential to evaluate the safety and efficiency of the service. The participants perceived the service to be safe and efficient for patients to continuing receiving the services. In addition, the managers were involved in evaluation of training and organisation of the service during the research project. This assists in adjusting the services to the local setting and improve routines throughout the project, through learning from prior mistakes. One municipal manager said: “*We have meetings going over how this is working and what can be done differently, you need to learn from your mistakes*.”

## Discussion

The service was experienced as valuable for the local inhabitants and tourists visiting the area. The municipal and the local medical centre managers were supportive of such a service in their community as it increases equality in health services for the rural population. In addition, the service was considered cost-effective to the society as unnecessary patient transfers were avoided and the number of patients needing comprehensive rehabilitation seemed reduced. Overall, the participants thought this service should be continued after the project ends.

This study showed that a remote-controlled stroke evaluation CT service could be set up using personnel on-site to keep the service operational also outside the normal dayshift. However, this requires task shifting and changing routines coordinated in several departments and across health care organisations. Considering that paramedics prepared the CT scanning, performed the patient examination as well as administered the required medication, the service required task shifting. A project manager from the internal medicine department was needed to plan routines, education, and training [8, 12]. The involvement of the radiology department especially the radiographers and a radiology project manager were vital to train the paramedics in CT procedures and radiation protection and to obtain permission from the radiation protection authority for the paramedics to handle patients undergoing CT-scan. This further required managers at the pre-hospital services to organise training sessions and changes in fleet management, showing the importance of support and flexibility in the organisation culture and management [13]. Using the remote controlled application, the contrast delay tracked does not function properly as the delays slow down the system. Thus, routines when performing the head CTA were changed. Subsequently the routine for the tertiary hospital serving the area also had to change, as they approved patient transfer without a CTA performed and interpreted beforehand. These changes in routines went way beyond the included departments and organisations. It required coordination and flexibility between and among the external organisations as well as within the included departments, these are important factors in successful innovation processes and especially when implementing telemedicine services [8, 10, 11].

According to May et al. [11] both normative and relational reconstruction are needed for implementation of new technologies to be successful and lasting. For continuation of the service after the project’s completion, the participants expressed the need for management support and coordination from all the involved departments. As the number of patients CT scanned after normal working hours was small, there would be a need for weekly training for all personnel groups also after the project ends. This would be possible only if the larger context adapted to and assimilated the new service into their routines, and management support is essential to achieve assimilation [10, 11]. In addition, knowledge on the cost-effectiveness of the service is required to determine whether the efforts produced the expected outcome for patients and society. According to Greenhalgh et al. [10] services that are cost-effective are more easily adopted into clinical practice. The clinical and socio-economic research accompanying this project was expected by the participants to provide evidence to enable the management at the hospital trust to decide whether to continue the service or not.

### Strength and limitations

The study used a qualitative inductive approach, this led to first hand experiences for the involved personnel and managers to be explored. However, a more theory driven approach could have prompted other topics to be discussed in the interviews.

This study has a combination of one focus group and several individual interviews. This was due to two factors. The ongoing covid-19 pandemic and difficulty in recruitment. Recruitment from the top management of the radiology department was difficult. The reason for this is unknown, this would have made a focus group from the hospital too small and unbalanced, resulting in individual interviews with managers from hospital. In addition, the pandemic made the gathering of people for a physical focus group meeting difficult. As an alternatively, several interviews were conducted online to reduce traveling and meeting in person. This combination may lead to a different depth in data obtained from managers in the focus group compared to managers and personnel in the individual interviews [23]. However, the mixing of individual and focus group interviews may also provide findings not possible if all interviews was either individual or in focus groups as the two methods have different strengths and limitations [23].

Patients and carers were not included in this study. Including patients and carers having experience from this service could have brought up further aspects and experiences, thus patients/cares should be explored in future studies.

The recruitment depended on the municipality and hospital management to identify potential volunteering participants. This may have resulted in volunteers responding positively to the project mainly to portray management in a favourable light. On the other hand, personnel who are judgemental towards the service could also be motivated to voluntarily participate and criticise the project anonymously. This study describes a project in one specific rural area of Norway. Thus, caution is warranted for transferability of the observed findings to other countries and contexts. However, rich, thick descriptions of the context, service set-up and participants would enable transferability assessment [24]. The results are based on self-reported data, no attempt have been made to verify their statements independently.

## Conclusions

This study showed that telemedicine stroke evaluation with a remote-controlled CT using local on call personnel was experienced as valuable in the local community, providing a sense of healthcare services security and equality. The set-up of the service required radiation protection and privacy approvals, which is important to be aware of when planning similar services. Task shifting for paramedics was a main task in the project. This supported the importance of a manager/coordinator role for education and training, as the paramedics needed to acquire theoretical and practical knowledge in working with the telemedicine application combined with CT scanning and new clinical tasks. Management involvement, flexibility, and a culture for coordination and cooperation both within and between the departments locally, and with external hospitals seems to be a key factor both in the implementation process and for keeping the service operating long term.

## Data Availability

The datasets generated and analysed during the current study are not publicly available due to participant anonymity issues. Dataset can be made available from the corresponding author on reasonable request.

## Abbreviations

CT: Computed tomography
CTA: Computed tomography angiography
NIHSS: National Institute of Health Stroke Scale
PACS: Picture archiving and communication system
RIS: Radiology information system
